# Cell type and receptor identity regulate cholera toxin subunit B (CTB) internalization

**DOI:** 10.1098/rsfs.2018.0076

**Published:** 2019-02-15

**Authors:** Anirudh Sethi, Amberlyn M. Wands, Marcel Mettlen, Soumya Krishnamurthy, Han Wu, Jennifer J. Kohler

**Affiliations:** 1 Department of Biochemistry, University of Texas Southwestern Medical Center, Dallas, TX 75390, USA; 2 Department of Cell Biology, University of Texas Southwestern Medical Center, Dallas, TX 75390, USA

**Keywords:** glycosylation, fucose, cholera, exotoxin, GM1

## Abstract

Cholera toxin (CT) is a secreted bacterial toxin that binds to glycoconjugate receptors on the surface of mammalian cells, enters mammalian cells through endocytic mechanisms and intoxicates mammalian cells by activating cytosolic adenylate cyclase. CT recognizes cell surface receptors through its B subunit (CTB). While the ganglioside GM1 has been historically described as the sole receptor, CTB is also capable of binding to fucosylated glycoconjugates, and fucosylated molecules have been shown to play a functional role in host cell intoxication by CT. Here, we use colonic epithelial and respiratory epithelial cell lines to examine how two types of CT receptors—gangliosides and fucosylated glycoconjugates—contribute to CTB internalization. We show that fucosylated glycoconjugates contribute to CTB binding to and internalization into host cells, even when the ganglioside GM1 is present. The contributions of the two classes of receptors to CTB internalization depend on cell type. Additionally, in a cell line that harbours both classes of receptors, gangliosides dictate the efficiency of CTB internalization. Together, the results lend support to the idea that fucosylated glycoconjugates play a functional role in CTB internalization, and suggest that CT internalization depends on both receptor identity and cell type.

## 1. Introduction

Cholera is caused by the pathogenic bacterium *Vibrio cholerae* [1]. *Vibrio cholerae* produces a protein toxin composed of A and B subunits, which form an AB_5_ complex. Cholera toxin (CT) binds to and invades host intestinal epithelial cells. Host cell surface molecules are recognized by the B subunit, facilitating cell entry by the A subunit, which activates adenylate cyclase, thereby leading to massive ion and fluid secretion. In the early 1970s, the ganglioside GM1 was identified as a high-affinity binding partner for cholera toxin subunit B (CTB) [2,3]. Further work showed that the addition of GM1 to CT-resistant cells confers susceptibility to intoxication [4,5]. The binding of CTB to the glycan headgroup of GM1 has been extensively characterized through various methods, demonstrating the interaction to be of high affinity with a nanomolar or picomolar *K*_d_ [6–8]. Further, structural analysis by X-ray crystallography has revealed the molecular details of CTB recognition of the GM1 glycan [9,10]. Based on this body of data, GM1 has been historically recognized as the sole receptor for CT [11]. Nonetheless, analysis of the glycosphingolipid composition of the normal human small intestinal epithelium, the physiological target for toxin action, revealed a surprisingly small amount of GM1 [12], calling into question the idea that GM1 is the sole functional receptor for CT. Further, B4galnt1-null mice lack GM1 but exhibit a stronger physiological response to CT than wild-type littermates, indicating that host GM1 is not required for CT action *in vivo* [13].

Epidemiological studies have implicated fucosylated ABO blood group antigens in determining the severity of cholera [14–17], and several reports showed that these blood group antigens could bind directly to different CTB variants [18,19]. We found that fucose (Fuc) is a key recognition determinant for CT binding to two human intestinal epithelial cell lines (T84 and Colo205): inhibition of fucosylation (using metabolic inhibitor 2-fluoro-peracetyl-fucose (2F-Fuc) [20]) dramatically reduces CTB binding to cells, largely blocks CTB entry into cells and reduces the ability of CT to raise intracellular cAMP levels, a key mechanistic step in host cell intoxication [21]. GM1-independent CT intoxication could be completely inhibited by brefeldin A, implying that this process relies on trafficking through the secretory pathway [13,21]. Additional experiments demonstrated a role for fucose in CTB binding to primary human epithelial cells [13,21], indicating that the cell culture results are unlikely to be an artefact of performing experiments in immortalized cell lines. Recognition of fucose by CTB was confirmed by co-crystal structures between CTB and difucosylated ABO blood group glycans, revealing a novel fucosylated glycan binding site distinct from the previously identified GM1 site [22,23], and by recent glycan array data that demonstrate CTB binding to biantennary, fucosylated human milk oligosaccharides (HMOs) [24]. Binding studies indicate that the interaction of CTB with fucosylated glycans has a much lower affinity than the CTB–GM1 interaction, with difucosylated blood group antigens exhibiting *K*_d_ values in the low millimolar range [19,22,23,25]. However, the functional significance of fucose recognition by CTB is underscored by observations that a variety of fucosylated molecules, as well as a fucose-recognizing lectin, competitively interfere with CTB binding to intestinal epithelial cell lines and primary cells [13,21,25].

With the knowledge that CT can use two classes of receptors—gangliosides and fucosylated glycoconjugates—we decided to evaluate how CT internalization is affected by receptor identity. We examined two cell lines that harbour different sets of endogenous CTB receptors. Using synthetic glycobiology approaches, we altered the receptor composition of each cell line and tested the effects on CTB binding and internalization. The results confirm that fucosylated glycoconjugates function in CTB binding and internalization, and demonstrate that internalization of CTB depends on both the receptor identity and the cellular context.

## 2. Results

### 2.1. Manipulation of cholera toxin subunit B receptor display through synthetic glycobiology

As described in detail below, we assessed CTB binding and internalization in two cell lines. First, we used T84 colonic epithelial cells, which are similar to the physiological target cells of CT and contain little endogenous GM1 [21]. Second, we used a respiratory epithelial cell line, HBEC3, which shares some characteristics with intestinal epithelial cells [26]. Cross-linking data imply that GM1 is present in HBEC3 cells [21]. Both T84 and HBEC3 cells produce fucosylated glycoconjugates. To evaluate the relative roles of gangliosides and fucosylated glycoconjugates in CTB internalization, we took a synthetic glycobiology approach, using small molecule reagents to inhibit biosynthesis of each class of receptors. To inhibit biosynthesis of gangliosides including GM1, we used NB-DGJ, which interferes with addition of glucose to ceramide and thereby blocks synthesis of gangliosides [27]. We have previously observed that 17 µg ml^−1^ NB-DGJ is sufficient to substantially reduce levels of glucosylceramide-based glycolipids in T84 cells [21]. To inhibit biosynthesis of fucosylated glycoconjugates, we used 2F-Fuc, a metabolic inhibitor that results in global fucosylation decrease [20]. When included in cell culture media at 200 µM, 2F-Fuc nearly eliminates binding of a fucose-recognizing lectin to glycoconjugates from T84 [21] and HBEC (data not shown) cells. We also increased the ganglioside levels in both cell types by exogenous addition of GM1. While we had some concern about whether exogenously added GM1 would incorporate correctly into the plasma membrane and function in the same way as endogenous GM1, a number of prior reports had shown that exogenously added GM1 is a functional receptor for CT [2,4,28]. We also attempted to increase fucosylation levels by culturing the cells in media supplemented with l-fucose [29], but did not observe an increase in fucosylation levels of the cell lines examined here. Therefore, we relied solely on inhibition of fucosylation with 2F-Fuc to evaluate the role of fucosylated glycoconjugates in CTB internalization.

### 2.2. Cholera toxin subunit B binds to both gangliosides and glycoproteins in lung epithelial cells

**Figure 1.**
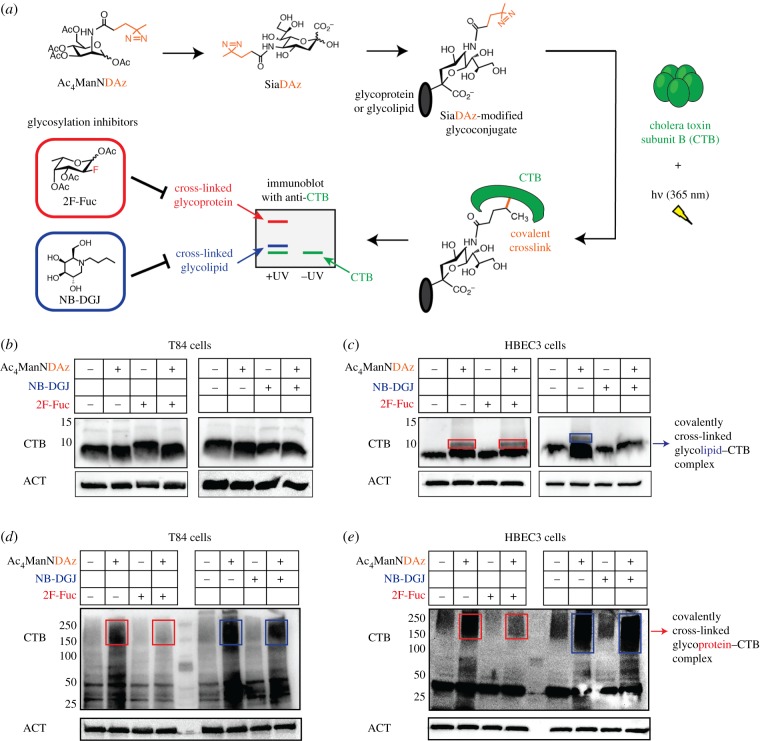
CTB cross-links to endogenous gangliosides in lung but not colonic epithelial cells. (*a*) Cells cultured with Ac_4_ManNDAz produce SiaDAz, a photocross-linking sugar that can be incorporated into glycoproteins and glycolipids in place of naturally occurring sialic acid. Cross-linking to fucosylated glycoproteins and to gangliosides can be assessed by evaluating the mobility of cross-linked CTB complexes by immunoblot, and by evaluating the sensitivity of cross-linking to inhibition by 2F-Fuc (for fucosylated glycoproteins) and NB-DGJ (for gangliosides). (*b*) T84 cells were cultured with Ac_4_ManNDAz or ethanol in the presence or absence of 200 µM 2F-Fuc or 40 µg ml^−1^ NB-DGJ for 3 days. Cells were then incubated with CTB, and UV irradiated. Cell lysates were analysed by 20% SDS–PAGE followed by immunoblot with anti-CTB antibody. β-Actin was used as a loading control. (*c*) The same as (*b*) but HBEC3 cells were used. Boxes highlight the effects of inhibitor (2F-Fuc or NB-DGJ) treatment on glycolipid–CTB cross-linking. (*d*,*e*) T84 and HBEC3 lysates from (*b*) and (*c*) were also analysed by 6% SDS–PAGE followed by immunoblot with anti-CTB antibody. Boxes highlight the effects of inhibitor (2F-Fuc or NB-DGJ) treatment on glycoprotein–CTB cross-linking. β-Actin was used as a loading control. Blots are representative images of two independent experiments. (Online version in colour.)

CTB binds to cell surface receptors at 4°C but is not internalized. To capture interactions between CTB and cell surface receptors, we used a cell-permeable precursor sugar (Ac_4_ManNDAz) that can be metabolized to a photocross-linking sialic acid analogue (SiaDAz) and incorporated into both glycoproteins and glycolipids in place of natural sialic acids [30]. Following UV irradiation, CTB cross-links to cell surface receptors that contain the modified SiaDAz (figure 1*a*) [21]. Because the cross-linker is attached to sialic acid, only sialylated receptors will be captured and any non-sialylated receptors will be invisible in this analysis. The cross-linked complexes can be separated by sodium dodecyl sulfate–polyacrylamide gel electrophoresis (SDS–PAGE) and visualized by CTB immunoblot. The size of the complex and its sensitivity to glycosylation inhibitors provides information about the identity of the glycoconjugate to which CTB is cross-linked. Prior work has shown that the species that migrates at approximately 13 kDa and is absent in cells cultured with NB-DGJ corresponds to CTB cross-linked to GM1 [31]. Similarly, higher molecular weight species that are reduced in intensity when cells are cultured with 2F-Fuc correspond to CTB cross-linked to fucosylated glycoproteins [13,21]. Here, we use SiaDAz cross-linking to assess types of CTB binding partners present in T84 and HBEC3 cells. As observed previously [21], no cross-linking of CTB to GM1 gangliosides was detected in T84 cells (figure 1*b*). However, CTB cross-linking to higher molecular weight glycoconjugates was observed, and was reduced when fucosylation was inhibited (figure 1*d*). As observed previously [21], two types of CTB cross-linked complexes were present in HBEC3 cells. The intensity of a lower molecular weight species was dramatically reduced when cells were cultured with NB-DGJ (figure 1*c*), suggesting that this band represents CTB cross-linked to gangliosides such as GM1, while the higher molecular weight complex was sensitive to 2F-Fuc treatment (figure 1*e*) and likely represents CTB cross-linked to glycoproteins, at least some of which are fucosylated. These data demonstrate that HBEC3 cells, unlike T84 cells, contain endogenous gangliosides that can bind to and cross-link to CTB. Further, inhibitors of glycosylation can be used to control the set of CTB receptors present in HBEC3 cells with NB-DGJ inhibiting ganglioside biosynthesis but having no effect on fucosylated glycoproteins, and 2F-Fuc inhibiting fucosylation but not ganglioside biosynthesis.

### 2.3. Gangliosides regulate cholera toxin subunit B internalization in lung epithelial cells

**Figure 2.**
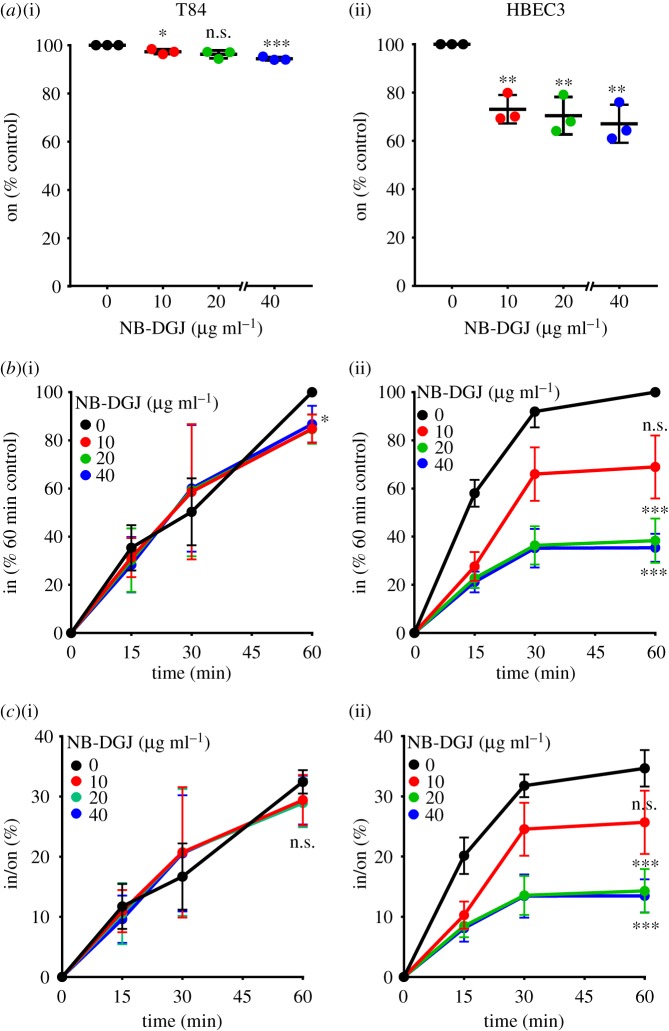
Endogenous gangliosides are significant contributors to CTB cell surface binding and internalization in lung but not colonic epithelial cells. (*a*–*c*) T84 and HBEC3 cells were cultured with the indicated concentrations of NB-DGJ (in µg ml^−1^) for 3 days, then incubated with 4 µg ml^−1^ CTB on ice for 30 min. (*a*) To measure cell surface binding of CTB by on-cell ELISA, cells were maintained at 4°C. The raw values obtained were averaged, normalized to the untreated control and compared with the untreated control for statistical analysis. (*b*) To measure internalization of CTB by in-cell ELISA, cells were incubated at 37°C for the indicated times and normalized to the values obtained for the untreated control at the 60 min time point. For statistical comparisons, internalization values at the 60 min time point were compared with the untreated control. (*c*) The efficiency of CTB internalization in T84 and HBEC3 cells was calculated by the ratio of internalized CTB at each of the time points in an individual experiment to the corresponding CTB cell surface binding values obtained by on-cell ELISA. For all panels, data represent three independent experiments performed on different dates. Each experiment comprised the average of four samples. Statistical significance determined by comparing data obtained from the three experiments by the unpaired Welch test: *** indicates *p* < 0.001, ** indicates *p* < 0.01, * indicates *p* < 0.05. n.s. indicates difference from the untreated sample not statistically significant. (Online version in colour.)

To enable CTB cell surface binding but not internalization, biotinylated CTB was added to cells on ice (4°C). CTB cell surface binding was measured by a modified cell-based enzyme-linked immunosorbent assay (ELISA) technique and was referred to as ‘on’ [32]. However, not all CTB binding events lead to cellular uptake [28]. Therefore, we also incubated cells at 37°C for a defined period of time to allow CTB internalization to occur. The amount of CTB internalized was measured by an in-cell ELISA method and was referred to as ‘in’ [32]. To test if endogenous gangliosides serve as functional CTB receptors, we measured the effect of NB-DGJ treatment on CTB binding and internalization. NB-DGJ treatment reduced CTB binding to HBEC3 cells significantly and to T84 cells minimally (figure 2*a*), consistent with CTB–ganglioside cross-linking observed in HBEC3 but not T84 cells (figure 1*b,c*). NB-DGJ treatment also reduced CTB internalization in HBEC3 cells in a concentration-dependent manner (figure 2*b*(ii)), but had minimal effect on CTB internalization in T84 cells (figure 2*b*(i)). We also calculated internalization efficiency, which is the fraction of bound CTB that is internalized (i.e. in/on) [33]. NB-DGJ treatment decreased CTB internalization efficiency in HBEC3 cells (figure 2*c*(ii)) but did not affect CTB internalization efficiency in T84 cells (figure 2*c*(i)). Thus, and in agreement with earlier studies [21], these data suggest that gangliosides do not contribute substantially to CTB cell surface binding or internalization in T84 cells. However, in HBEC3 cells, gangliosides are important contributors to CTB cell surface binding and internalization, and CTB internalization efficiency decreases when gangliosides are absent.

### 2.4. Fucosylation regulates cholera toxin subunit B binding and internalization, even in the presence of endogenous gangliosides

We have shown that the inhibition of fucosylation (using the metabolic inhibitor 2F-Fuc) results in dramatic reductions in CTB binding to and internalization in T84 cells [21], implying that fucosylated glycoconjugates act as CTB receptors. With the observation that CTB cross-links to both gangliosides and fucosylated glycoproteins in HBEC3 cells (figure 1*c,e*), we wanted to test how the inhibition of fucosylation affected CTB binding to and internalization in cells that contain endogenous gangliosides.

**Figure 3.**
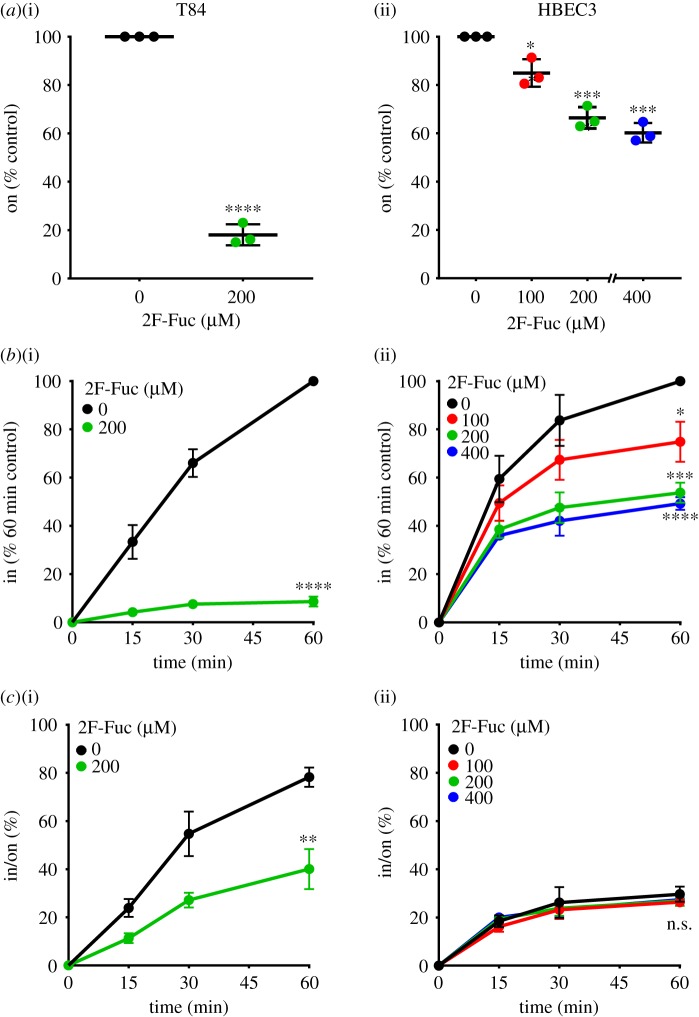
Fucosylation regulates CTB cell surface binding and internalization in both colonic and lung epithelial cells. T84 and HBEC3 cells were cultured with the indicated concentrations of 2F-Fuc (in µM) for 3 days, then incubated with 4 µg ml^−1^ CTB on ice for 30 min. (*a*) To measure cell surface binding of CTB by on-cell ELISA, cells were maintained at 4°C. The raw values obtained were averaged, normalized to the untreated control and compared with the untreated control for statistical analysis. (*b*) To measure internalization of CTB by in-cell ELISA, cells were incubated at 37°C for the indicated times and normalized to the values obtained for the untreated control at the 60 min time point. For statistical comparisons, internalization values at the 60 min time point were compared with the untreated control. (*c*) The efficiency of CTB internalization in T84 and HBEC3 cells was calculated by the ratio of internalized CTB at each of the time points in an individual experiment to the corresponding CTB cell surface binding values obtained by on-cell ELISA. The data represent three independent experiments performed on different dates. Statistical significance determined by the unpaired Welch test: **** indicates *p* < 0.0001, *** indicates *p* < 0.001, ** indicates *p* < 0.01, * indicates *p* < 0.05. n.s. indicates difference from the untreated control not statistically significant. (Online version in colour.)

As reported previously [21], T84 cells cultured with 200 µM 2F-Fuc showed a robust decrease in CTB cell surface binding and internalization (figure 3*a*(i)*,b*(i)). In HBEC3 cells, 2F-Fuc also reduced CTB cell surface binding and internalization in a concentration-dependent manner (figure 3*a*(ii)*,b*(ii)). While the inhibition of fucosylation resulted in decreased CTB internalization efficiency in T84 cells (figure 3*c*(i)), it had no meaningful effect on CTB internalization efficiency in HBEC3 cells (figure 3*c*(ii)). Taken together, the results in figures 2 and 3 show that gangliosides control the efficiency of CTB endocytosis in HBEC3 cells (even in the presence of fucosylated receptors) and that fucosylation determines the efficiency of CTB endocytosis in T84 cells (which lack measurable quantities of CTB binding gangliosides).

### 2.5. Exogenous GM1 is a functional cholera toxin receptor

**Figure 4.**
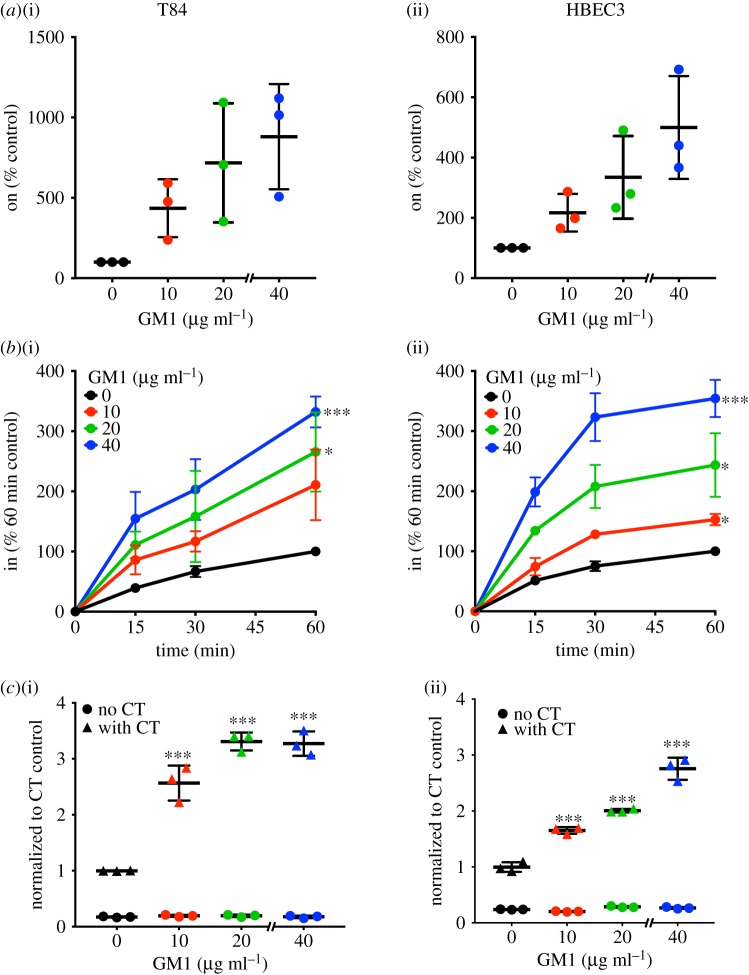
Exogenously added GM1 promotes CTB binding and internalization in both colonic and lung epithelial cells. (*a*) T84 or HBEC3 cells were incubated with the indicated concentrations of GM1 at 4°C, then CTB binding was measured by on-cell ELISA (T84 and HBEC3). The raw values obtained were averaged, normalized to the untreated control and compared with the untreated control for statistical analysis. (*b*) To measure internalization of CTB by in-cell ELISA, cells were incubated at 37°C for the indicated times and normalized to the values obtained for the untreated control at the 60 min time point. For statistical comparisons, internalization values at the 60 min time point were compared with the untreated control. Data in (*a*) and (*b*) represent three independent experiments performed on different dates. (*c*) T84 and HBEC3 cells were treated with the indicated concentrations of GM1, followed by exposure to phosphate-buffered saline (filled circle) or 0.1 nM CT holotoxin (filled triangle) for 1 h. cAMP levels were measured by ELISA. The cAMP levels for CT-treated samples are reported relative to the untreated cells. The cAMP levels for non-CT-treated samples are reported relative to CT-treated and vehicle-treated cells. Data shown represent an average of four replicate samples. A replicate experiment yielded similar results. Statistical significance determined by the unpaired Welch test: **** indicates *p* < 0.0001, *** indicates *p* < 0.001, ** indicates *p* < 0.01, * indicates *p* < 0.05. n.s. indicates difference not statistically significant. (Online version in colour.)

We wondered whether fucosylation determines endocytic efficiency in T84 cells simply because they lack gangliosides like GM1 [21]. Exogenously added GM1 can be incorporated into the plasma membrane of cells and results in increased sensitivity of cells to the toxin [2,4,34]. We next asked whether exogenously added GM1 could control the efficiency of CTB endocytosis in either or both cell lines. Upon adding GM1 exogenously, we observed that CTB cell surface binding increased in both T84 and HBEC3 cells in a concentration-dependent manner (figure 4*a*). At the highest GM1 concentration (40 µg ml^−1^), we observed an approximately 10-fold increase in CTB binding to T84 cells and an approximately fivefold increase in CTB binding to HBEC3 cells.

Unfortunately, GM1 can adhere to the cell culture dishes in the absence of cells (data not shown). Therefore, some fraction of the observed CTB binding (figure 4*a*) could be due to CTB binding to GM1 adhered to the cell culture plate. However, GM1 treatment also resulted in increased CTB internalization in both T84 and HBEC3 cells (figure 4*b*). If any GM1 adhered to the plate, it was removed in the acid wash step of the in-cell ELISA or not detected because the CTB–biotin bound to it is blocked with avidin. Therefore, the observed internalization (figure 4*b*) can be fully attributed to GM1 that has incorporated into plasma membranes of cells. Nonetheless because we could not ascertain with certainty the amount of CTB that was binding to the cell culture dish versus cells (even with complete cell confluency), we decided not to calculate CTB internalization efficiency in the case of GM1 treatment. To further assess whether CTB internalization promoted by exogenously added GM1 was functionally relevant, we measured the ability of CT to raise intracellular cAMP levels under these conditions. In both T84 and HBEC3 cells, GM1 treatment increased the CT-induced cAMP accumulation, implying that GM1 promotes CTB internalization that is on-pathway to host cell intoxication (figure 4*c*). Taken together, these results demonstrate that at least some fraction of exogenously added GM1 inserts in the plasma membrane, where it acts as a functional receptor that promotes CTB internalization and CT host cell intoxication in both cell types.

### 2.6. Fucosylated glycans contribute to cholera toxin subunit B internalization, even in the presence of GM1

**Figure 5.**
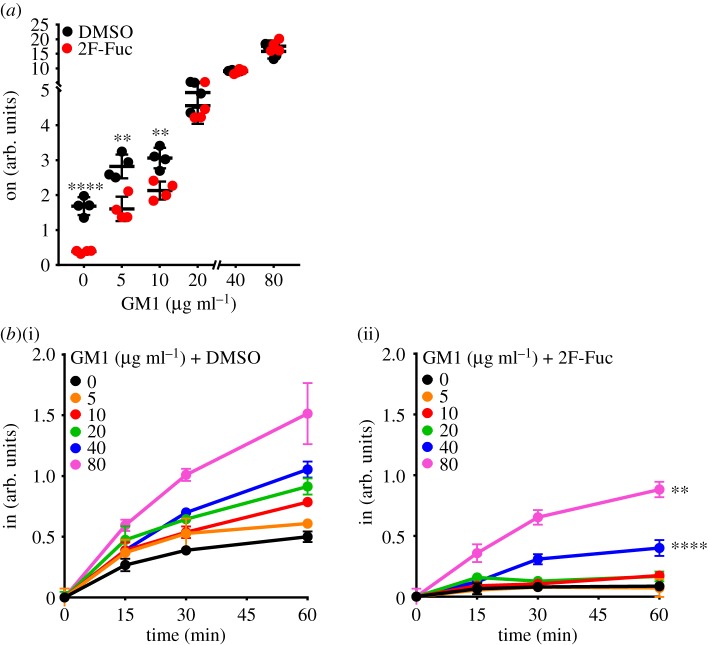
Fucosylation regulates CTB cell surface binding and internalization in colonic epithelial cells, even in the presence of GM1. (*a*) T84 cells were cultured with 200 µM 2F-Fuc or dimethyl sulfoxide (DMSO) for 3 days, then incubated with indicated concentrations of GM1 for 30 min, followed by 4 µg ml^−1^ CTB on ice for 30 min. The binding of CTB was measured by on-cell ELISA. The raw values obtained were averaged, normalized to the untreated control and compared with the untreated control for statistical analysis. (*b*) T84 cells were cultured with DMSO (i) or 200 µM 2F-Fuc (ii) for 3 days, then incubated with the indicated concentrations of GM1 for 30 min, followed by 4 µg ml^−1^ CTB on ice for 30 min, followed by incubation at 37°C for measuring CTB internalization at the indicated time points, and normalized to the values obtained for the untreated control at the 60 min time point. The internalization of CTB was measured by in-cell ELISA. For statistical comparisons, internalization values at the 60 min time point were used. The 40 and 80 µg ml^−1^ GM1 treated with 2F-Fuc were compared with the respective samples without 2F-Fuc treatment for statistical analyses. Statistical significance determined by the unpaired Welch test: **** indicates *p* < 0.0001, *** indicates *p* < 0.001, ** indicates *p* < 0.01, * indicates *p* < 0.05. n.s. indicates difference not statistically significant. (Online version in colour.)

Having observed that exogenously added GM1 resulted in increased CTB cell surface binding and internalization in T84 cells, we next interrogated the role that endogenous receptors play in controlling CTB binding and internalization in these cells. We used 2F-Fuc treatment to create T84 cells where endogenous fucosylated receptors were either present or absent, and then added increasing concentrations of GM1 to both. As observed previously (figure 3*a*) [21], 2F-Fuc treatment results in decreased CTB binding; this decrease was still observed in the presence of low concentrations (5, 10 µg ml^−1^) of GM1, but was eliminated at higher (20, 40, 80 µg ml^−1^) GM1 concentrations (figure 5*a*). Thus, at low GM1 concentrations, both fucosylated glycoconjugates and GM1 contribute to CTB binding. However, at high GM1 concentrations, GM1 is the dominant binding partner, consistent with the higher affinity of CTB for GM1 when compared with fucosylated structures [7,19,22,23,25,35,36]. We also measured CTB internalization under these conditions. As observed previously [21], in the absence of exogenous GM1, the inhibition of fucosylation nearly eliminated CTB internalization (figures 3*c* and 5*b*). The inhibition of fucosylation also significantly reduced CTB internalization when exogenous GM1 was added, even at the highest GM1 concentrations—40 and 80 µg ml^−1^ (figure 5*b*). Taken together, these data indicate that CTB internalization in T84 cells is at least partially regulated by endogenous fucosylated glycoconjugates, even when the high-affinity GM1 receptor is present.

### 2.7. Gangliosides and fucosylated glycoconjugates are not the only cholera toxin subunit B receptors

**Figure 6.**
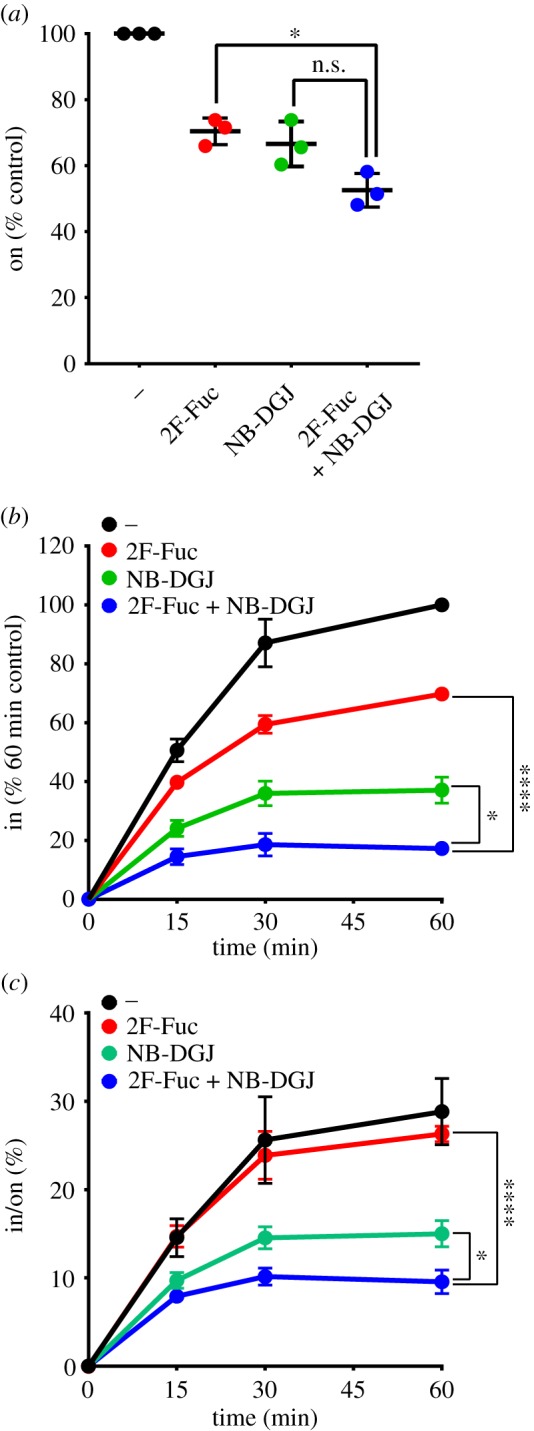
Inhibiting fucosylation or gangliosides does not completely block CTB cell surface binding and internalization in lung epithelial cells. (*a*–*c*) HBEC3 cells were cultured with 200 µM 2F-Fuc and 40 µg ml^−1^ NB-DGJ for 3 days, then incubated with 4 µg ml^−1^ CTB on ice for 30 min. (*a*) To measure cell surface binding of CTB by on-cell ELISA, cells were maintained at 4°C. The raw values obtained were averaged, and normalized to the untreated control. (*b*) To measure internalization of CTB by in-cell ELISA, cells were incubated at 37°C for the indicated times and normalized to the values obtained for the untreated control at the 60 min time point. For statistical comparisons, internalization values at the 60 min time point were used. (*c*) The efficiency of CTB internalization in HBEC3 cells was calculated by the ratio of internalized CTB at each of the time points in an individual experiment to the corresponding CTB cell surface binding values obtained by on-cell ELISA. The data represent three independent experiments performed on different dates. Statistical significance determined by the unpaired Welch test: **** indicates *p* < 0.0001, * indicates *p* < 0.05. n.s. indicates difference not statistically significant. (Online version in colour.)

We next wondered if fucosylated glycoconjugates and gangliosides are the only CTB receptors. To test this idea, we treated HBEC3 cells with concentrations of NB-DGJ and 2F-Fuc that had yielded maximal inhibitory effects on CTB binding (figures 2 and 3). NB-DGJ and 2F-Fuc individually blocked CTB cell surface binding and internalization (figure 6*a,b*). Co-treatment with both inhibitors did not result in a statistically significant decrease in CTB when compared with either single inhibitor treatment (figure 6*a*). However, CTB internalization was further decreased by co-treatment with NB-DGJ and 2F-Fuc, when compared with the individual treatments (figure 6*b*). While 2F-Fuc by itself did not reduce CTB internalization efficiency, cells treated with both NB-DGJ and 2F-Fuc displayed less efficient CTB internalization when compared with cells treated with NB-DGJ alone (figure 6*c*). Thus, when both types of receptors are present, gangliosides appear to play the dominant role in determining the efficiency of CTB internalization. Nonetheless, even with NB-DGJ and 2F-Fuc co-treatment, CTB internalization was not completely blocked, suggesting that functional CTB receptors may remain present on the surface of these cells.

## 3. Discussion

Here, we investigated the contributions of different classes of receptors to CTB internalization in two cell lines. Our experimental design allowed us to independently assess the roles of gangliosides and fucosylated glycoconjugates, albeit with some limitations. The metabolic inhibitor 2F-Fuc allowed us to eliminate fucosylated receptors. NB-DGJ treatment reduced ganglioside production in HBEC3 cells, but it was difficult to assess the effect of NB-DGJ in T84 cells because we cannot easily detect GM1 in these cells. We were able to increase ganglioside levels in both cell lines through the addition of exogenous GM1. We showed that this added GM1 was a functional receptor for CT, although we cannot exclude the possibility that some fraction of the added GM1 behaves differently from endogenous GM1. Despite this limitation, we determined that fucosylated glycoconjugates are important mediators of CTB endocytosis in T84 cells, even in the presence of GM1. Additionally, in HBEC3 cells, we were able to assess how the two classes of inhibitors contribute to CTB internalization efficiency. In this cell type, both gangliosides and fucosylated glycoconjugates contribute to CTB internalization with gangliosides playing a more important role in determining the efficiency of CTB internalization (figures 2*c*, 3*c* and 6*c*). If gangliosides and fucosylated glycoconjugates are acting as independent receptors, this result would suggest that gangliosides are more efficient at internalizing CTB than fucosylated glycoconjugates are. It is noteworthy that GM1 is a high-affinity CTB binder, while the fucosylated ligands for CTB thus far identified are much lower affinity [22,23,25]. Nonetheless, CTB binding studies demonstrate that low-affinity ligands can contribute to recognition even in the presence of a much higher affinity ligand [37].

The results presented here show that CTB can enter T84 cells (which have little GM1) as well as HBEC3 cells treated with NB-DGJ to reduce GM1 levels. These results lend additional support to the idea that fucosylated glycoconjugates function as receptors that mediate host cell intoxication by CT [21]. It remains possible that T84 cells and HBEC3 cells treated with NB-DGJ each contain some low level of GM1. We did not perform a genetic knockout of GM1 biosynthesis here, but studies in mice lacking B4galnt1 (an enzyme required for GM1 biosynthesis) demonstrate that GM1 is not required for intoxication by CT [13]. We also observed that fucosylated structures can contribute to CTB binding even when the high-affinity receptor GM1 is present (figure 3*a*(ii) and 5*a*). These results imply that caution should be applied when using CTB to detect the presence or localization of GM1, as the expression level of both GM1 and other CTB binding partners will vary among different cell lines [38–40]. In T84 cells, the addition of exogenous GM1 resulted in a strong increase in CTB cell surface binding but only a modest increase in CTB internalization. A possible interpretation of this result is that the fucosylated glycoconjugates present in these cells could be efficient CTB receptors. Alternatively, fucosylated glycoconjugates and GM1 may work together in CTB internalization, by acting either in concert or sequentially. Finally, although we showed that exogenously added GM1 is a functional receptor (figure 4*c*), this added GM1 could behave differently from endogenously produced GM1. Indeed, GM1 can also be produced in the course of cholera infection through the action of *V. cholerae* neuraminidase, which removes sialic acid from glycoconjugates in intestinal epithelial cells, thereby revealing endogenous GM1 [41,42]. Future studies will assess how GM1 produced through *V. cholerae* neuraminidase treatment affects the CTB internalization.

The critical role for fucosylated glycoconjugates in CTB internalization is supported by the cross-linking studies (figure 1*d,e*) and by the effects of 2F-Fuc on CTB internalization (figures 3 and 5). We observed fucose-dependent cross-linking of CTB to high molecular weight species, which are assumed to be glycoproteins. However, fucosylated glycoconjugates that lack sialic acid would not be observed in the cross-linking analysis, but could contribute to the changes in CTB binding and internalization observed with 2F-Fuc treatment. Therefore, additional fucosylated glycoproteins or fucosylated glycolipids may be present in these cells. We postulate that the fucosylated CTB receptor might not be a single molecule, but could represent a diverse set of molecules that exhibit distinct CTB binding and internalization characteristics. In future work, we aim to identify these distinct fucosylated species and assess their contributions to CTB endocytosis and to host cell intoxication by CT. Further, in HBEC3 cells, measurable CTB binding and internalization occurred even with co-treatment with NB-DGJ and 2F-Fuc. This result could reflect the incomplete inhibition of fucosylated glycoconjugate and/or ganglioside biosynthesis. Alternatively, the residual internalization may suggest the existence of an additional class of non-ganglioside, non-fucosylated CT receptors, consistent with our prior studies of mouse intestinal epithelial cells [13]. Thus, CT, like other bacterial toxins [43–45], may have evolved to exploit multiple host receptors.

## 4. Material and methods

### 4.1. General chemicals

Monosialoganglioside GM1 NH_4_^+^ salt (GM1) was purchased from Matreya (State College, PA, USA) (catalogue no. 1061); stock concentrations were made at 1 mg ml^−1^ in methanol. Previously synthesized very good purity grade Ac_4_ManNDAz was used [30,46]. Dimethyl sulfoxide (DMSO) was purchased from Sigma (St. Louis, MO, USA) (catalogue no. D2650). *N*-(*n*-butyl)deoxygalactonojirimycin (NB-DGJ; 98% pure) was purchased from Santa Cruz Biotechnology (Dallas, TX, USA) (catalogue no. sc-221974); stock concentrations were made at 5 mg ml^−1^ in water then sterile filtered. 2-Fluoro-peracetyl-fucose (2F-Fuc; 98.8% pure) was purchased from EMD Millipore (Darmstadt, Germany) (catalogue no. 344827); stock concentrations were made at 200 mM in DMSO. Bovine serum albumin (BSA) was purchased from Sigma (catalogue no. A9647). Paraformaldehyde (formaldehyde) aqueous solution (20%) was purchased from Electron Microscopy Sciences (Hatfield, PA, USA) (catalogue no. 15713). CTB used for photocross-linking experiments was purchased from Sigma (catalogue no. C9903). Biotin-conjugated CTB used for binding and internalization experiments was purchased from Thermo-Fisher Scientific (catalogue no. C-34779). CT (azide-free) from *V. cholerae* used for cAMP experiments was purchased from List Biological Laboratories (Campbell, CA, USA) (catalogue no. 100B).

### 4.2. Antibodies

The sources of the antibodies used for immunoblotting are as follows: anti-CT antibody (Sigma, catalogue no. C3062) and anti-β-actin antibody (Cell Signaling Technology, catalogue no. 3700). Goat anti-rabbit immunoglobulin G–horseradish peroxidase (IgG-HRP) conjugate (catalogue no. 65-6120) and goat anti-mouse IgG-HRP conjugate (catalogue no. 62-6520) secondary antibodies were purchased from Thermo-Fisher Scientific.

### 4.3. Cell culture

The following reagents for general cell culture use were purchased from Thermo-Fisher Scientific/Gibco (Carlsbad, CA, USA): Dulbecco's modified Eagle's medium (DMEM)/F-12 medium supplemented with 2.5 mM l-glutamine, 15 mM HEPES (catalogue no. 11330032), penicillin–streptomycin (P/S) (catalogue no. 15140122), fetal bovine serum (FBS) (catalogue no. 16000044), TrypLE express enzyme with phenol red (catalogue no. 12605010) and 1 M HEPES (catalogue no. 15630080). Dulbecco's phosphate-buffered saline (PBS) was purchased from Sigma (catalogue no. D8537). T84 cells (ATCC, Manassas, VA, USA) were maintained in DMEM/F-12 medium, 5% FBS, 1% HEPES and 1% P/S. Human bronchial epithelial cells (HBEC3) were obtained from John Minna, UT Southwestern Medical Center, and were maintained in EpiCM medium supplemented with 2% FBS, 1% epithelial cell growth support (EpiCGS), 1% P/S (ScienCell Research Labs, Carlsbad, CA, USA) (catalogue no. 4101). The cell lines were maintained at 37°C, 5% carbon dioxide in a water-saturated environment. The Countess automated cell counter (Life Technologies) was used for cell counting.

### 4.4. SiaDAz-mediated cholera toxin subunit B cross-linking

For photocross-linking of CTB to T84 or HBEC3 cells: 250 000 cells were seeded in 2 ml of medium into two separate six-well tissue culture plates (for –/+UV) that were pre-treated 10–15 min before cells were seeded with either vehicle (ethanol) or 100 µM Ac_4_ManNDAz until the vehicle evaporated. Cells were seeded with 10 µg ml^−1^ NB-DGJ and 200 µM 2F-Fuc or their vehicle control (water for NB-DGJ and DMSO for 2F-Fuc). After culturing for 72 h, the medium in each well was replaced with 1 ml fresh medium containing approximately 4.5 µg of CTB (Sigma) for 45 min at 4°C in the dark. The cells were then either kept at 4°C for an additional 45 min (for –UV samples) or irradiated on an ice/water bath (at approx. 4°C) for 45 min (for +UV samples) at 365 nm. Wells were washed twice with PBS, lysed using radioimmunoprecipitation assay buffer and incubated on ice for 30–60 min. The lysate was centrifuged at 21 000*g* for 10 min at 4°C to remove insoluble debris, and the supernatant was retained for separation on both a higher (15–20%) and lower (6%) percentage polyacrylamide gel. The samples were then transferred to a polyvinylidene difluoride (PVDF) membrane, and the blots were probed overnight at 4°C for anti-CT (Sigma; 1 : 10 000 dilution). Membranes were re-probed for the loading control anti-β-actin (Cell Signaling Technology; 1 : 5000 dilution).

### 4.5. Immunoblot

Twenty micrograms of T84 and HBEC3 cell lysates were separated by SDS–PAGE. After overnight transfer to PVDF membrane, the membrane was blocked with Tris-buffered saline buffer with 0.1% Tween-20 (TBST) containing 3% BSA at room temperature for 1 h, followed by incubation with primary antibodies (diluted with TBST containing 3% BSA) on a rocker at 4°C overnight. After three washes with TBST, the membrane was incubated with the relevant secondary antibodies (goat anti-mouse or goat anti-rabbit; 1 : 10 000 dilution) in TBST containing 1% BSA for 1 h. The membranes were washed three times with TBST and then images were developed with SuperSignal West Femto Maximum Sensitivity Substrate (Thermo-Fisher Scientific, catalogue no. 34095) for 1 min, then imaged with a ChemiDoc MP Imaging system (Bio-Rad, Hercules, CA, USA).

### 4.6. Cholera toxin subunit B cell surface binding assay (on-cell ELISA)

T84 and HBEC3 cells (25 000/well) were cultured in medium in the absence or presence of each inhibitor in individual wells of a 96-well plate (Costar, catalogue no. 9102) for 3 days. In a typical experiment, four samples (wells) were prepared for each condition. On the day of the experiment, cells were washed three times in cold PBS, and further incubated with 4 µg ml^−1^ of biotinylated CTB in PBS4+ (1 mM CaCl_2_, 1 mM MgCl_2_, 0.2% (w/v) BSA and 5 mM glucose) for 30 min on ice. Unbound biotin–CTB was washed away three times in cold PBS. Then cells were fixed with 4% paraformaldehyde for 10 min on ice and 20 min at room temperature. After three washes with PBS, cells were blocked for 20 min with Q-PBS (PBS supplemented with 0.01% (w/v) saponin, 2% (w/v) BSA and 0.1% (w/v) lysine, pH 7.4). The cells were then incubated at room temperature for 1 h in streptavidin–HRP (1 : 10 000; Roche) conjugate diluted in Q-PBS. HRP activity was measured by a stopped colorimetric assay using ortho-phenylenediamine as a substrate. Light absorption at 490 nm was determined with a Synergy Neo microplate reader (BioTek, Winooski, VT, USA) and all values were corrected by light absorbance at 650 nm and normalized by total cell protein content (bicinchoninic acid assay; BCA protein assay kit; Pierce). Four replicate samples from a single experiment were averaged together. Data presented represent three experiments (four replicates each) performed on separate days.

### 4.7. Cholera toxin subunit B internalization assay (in-cell ELISA)

CTB internalization was measured by the on-cell ELISA, described above, with the following adaptations. During the experiment, control samples (to measure total surface-bound biotin–CTB) were washed three times with ice-cold PBS, then kept on ice awaiting analysis. Experimental samples were warmed to 37°C for the indicated times (0, 15, 30 and 60 min) to allow endocytic uptake, then endocytosis was halted by returning cells to ice and washing three times with cold PBS. Non-internalized biotinylated CTB was masked by successive treatment with 50 µg ml^−1^ of avidin (Sigma-Aldrich) for 1 h on ice, followed by three 1 min cold acid washes (0.2 M acetic acid/0.2 M NaCl). Cells were then washed six times with cold PBS and were fixed with 4% paraformaldehyde and further permeabilized with 0.1% (v/v) Triton X-100 in PBS for 15 min. After three washes with PBS, cells were blocked for 20 min with Q-PBS. Cells were then incubated at room temperature for 1 h in streptavidin–HRP conjugate diluted in Q-PBS. Reactive aldehydes and non-specific binding sites were quenched with Q-PBS. HRP activity was measured as described above. To combine data from multiple experiments, we normalized the ‘in’ values to the control 60 min internalization time point.

### 4.8. Cholera toxin subunit B internalization efficiency (in/on)

To measure CTB internalization efficiency, the fraction of internalized CTB (at 37°C) relative to the initial total surface bound ligand at 4°C (without the acid wash step) was calculated (both cell surface binding and internalization were measured in parallel for all the assays). At the end of CTB binding and internalization assays, the corrected data obtained by colorimetric assay (measured at 490 nm and corrected with the 650 nm data) were normalized to the total protein content measured by the bicinchoninic acid assay. The values obtained in cells incubated at time 0 were subtracted from the four time points (0, 15, 30 and 60 min). The resultant values provided the ratio or efficiency of CTB internalized at a given time point to the amount of CTB bound to the cell surface.

### 4.9. cAMP measurement

One hundred microlitres of 50 000 cells ml^−1^ cell suspensions of individual cell lines were added in wells in white-walled 96-well plates (Thermo-Fisher Scientific, catalogue no. 07-200-628) and cultured for 72 h. The wells were washed twice with PBS and then treated with 0.1 nM CT holotoxin at 37°C for 60 min (List Biologicals) in complete induction buffer provided in the cAMP-Glo kit (Promega, catalogue no. V1501). The assay was performed following the manufacturer's protocol (cAMP-Glo assay; Promega). The luminescence values were obtained using a Synergy Neo microplate reader (BioTek, Winooski, VT, USA).

## Data accessibility

This article has no additional data.
